# A Transposon in *Comt* Generates mRNA Variants and Causes Widespread Expression and Behavioral Differences among Mice

**DOI:** 10.1371/journal.pone.0012181

**Published:** 2010-08-17

**Authors:** Zhengsheng Li, Megan K. Mulligan, Xusheng Wang, Michael F. Miles, Lu Lu, Robert W. Williams

**Affiliations:** 1 Department of Anatomy and Neurobiology, Center for Integrative and Translational Genomics, University of Tennessee Health Science Center, Memphis, Tennessee, United States of America; 2 Department of Pharmacology and Toxicology, Virginia Commonwealth University, Richmond, Virginia, United States of America; Deutsches Krebsforschungszentrum, Germany

## Abstract

**Background:**

Catechol-O-methyltransferase (COMT) is a key enzyme responsible for the degradation of dopamine and norepinephrine. COMT activity influences cognitive and emotional states in humans and aggression and drug responses in mice. This study identifies the key sequence variant that leads to differences in *Comt* mRNA and protein levels among mice, and that modulates synaptic function and pharmacological and behavioral traits.

**Methodology/Principal Findings:**

We examined *Comt* expression in multiple tissues in over 100 diverse strains and several genetic crosses. Differences in expression map back to *Comt* and are generated by a 230 nt insertion of a B2 short interspersed element (B2 SINE) in the proximal 3′ UTR of *Comt* in C57BL/6J. This transposon introduces a premature polyadenylation signal and creates a short 3′ UTR isoform. The B2 SINE is shared by a subset of strains, including C57BL/6J, A/J, BALB/cByJ, and AKR/J, but is absent in others, including DBA/2J, FVB/NJ, SJL/J, and wild subspecies. The short isoform is associated with increased protein expression in prefrontal cortex and hippocampus relative to the longer ancestral isoform. The *Comt* variant causes downstream differences in the expression of genes involved in synaptic function, and also modulates phenotypes such as dopamine D1 and D2 receptor binding and pharmacological responses to haloperidol.

**Conclusions/Significance:**

We have precisely defined the B2 SINE as the source of variation in *Comt* and demonstrated that a transposon in a 3′ UTR can alter mRNA isoform use and modulate behavior. The recent fixation of the variant in a subset of strains may have contributed to the rapid divergence of inbred strains.

## Introduction

Degradation of the catecholamine neurotransmitters dopamine, epinephrine, and norepinephrine is catalyzed by catechol-O-methyltransferase (COMT) and the monoamine oxidase enzymes. The mechanism of transmitter inactivation is distinct for each enzyme—methylation for COMT and deamination for monamine oxidase—and the impact each exerts on neurotransmitter tone varies by brain region. COMT is a key enzyme regulating dopamine tone in frontal neocortex in which expression is high relative to the monoamine oxidases and dopamine transporters, but COMT contributes less to dopamine tone in the striatum and nucleus accumbens [1], [2], [3], [4]. Dopamine levels play a critical role in the modulation of cognitive and emotional states, and changes in dopamine signaling are hallmarks of many psychiatric and addiction disorders. In humans, non-human primates, and rodents dopamine levels and cognitive performance have been described by an inverted U-shaped relation in which too much or too little extracellular dopamine has detrimental effects [5], [6], [7].

Multiple SNPs have been identified that lead to changes in mRNA, protein, and/or activity levels but a single allele (Val108/158Met) accounts for most of the variation in the level of COMT activity in humans. Individuals homozygous for the Met allele have a 3- to 4-fold reduction in COMT activity and increased prefrontal cortex dopamine levels [8]. Although mice lack this particular polymorphism, several recent studies have shown that variation in *Comt* mRNA level [9] and activity [10] between C57BL/6J (B6) and DBA/2J (D2) mouse strains is associated with differences in aggression level and morphine sensitivity, respectively.

The B6 and D2 strains differ greatly on a wide variety of traits and their genomes are highly polymorphic with approximately 5 million identified sequence variants (www.sanger.ac.uk and www.genenetwork.org). A genetic reference population of B6 X D2 (BXD) recombinant inbred lines made by crossing these parental strains has been used extensively to explore differences in gene expression, neuropharmacology, and behavior, often using quantitative trait locus (QTL) mapping methods [11], [12]. The expression of *Comt* in this family of strains varies over 2-fold in several CNS regions and is strongly modulated by a polymorphism located within *Comt* itself [13]. However, there are no known SNPs between B6 and D2 in *Comt*, and the gene is situated in a ∼8 Mb region that is identical by descent among most inbred strains. The genetic mechanism of the observed variation in *Comt* expression has been an unresolved question. In this paper we report the cloning of the *cis* sequence variant that controls *Comt* mRNA expression in many inbred strains and that produces widespread effects in the BXD family. The cause of this *Comt cis*-regulation is the insertion of a strong and premature polyadenylation signal in the proximal 3′ UTR in B6 and several other strains. As a consequence these strains produce short 3′UTRs compared to strains without the insertion. This is the first known variant in mice that causes variation in *Comt* expression. This mutation has effects on COMT activity and is clearly linked to downstream variation in expression of many other genes, dopamine tone, and behavior. We find both expected and novel biological roles for *Comt* that are relevant for the diagnosis and treatment of psychiatric and cognitive diseases in humans.

## Results

### Variation in *Comt* mRNA expression and *cis*-regulation in CNS and peripheral tissues

**Figure 1:**
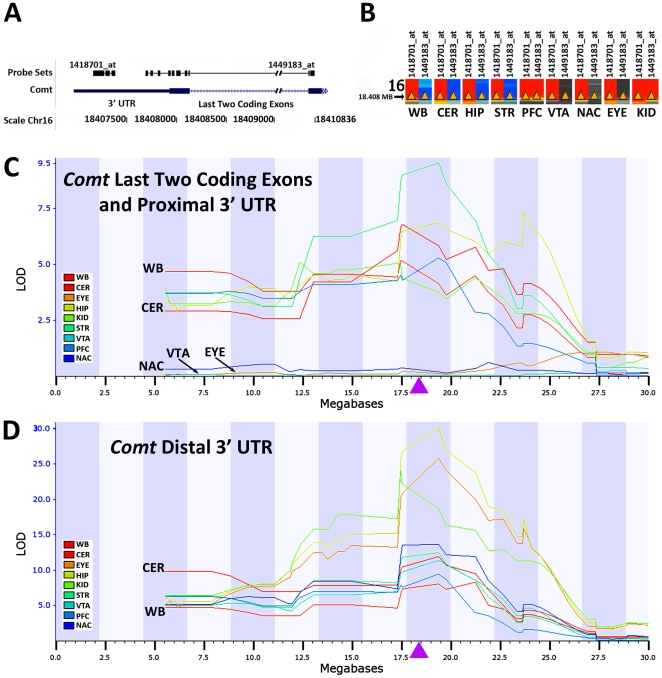
*Cis* modulation of *Comt* expression by *B* and *D* alleles in different tissues. (A) Orientation of probe sets relative to the *Comt* gene based on the UCSC Genome Browser Mouse July 2007 Assembly (mm9). Probe sets 1418701_at and 1449183_at are associated with mRNA containing distal 3′ UTR and the last two coding exons and proximal 3′ UTR of *Comt* mRNA, respectively. (B) *Cis*-modulation of *Comt* expression visualized using the QTL heatmap tool available at www.genenetwork.org. Coloration at 18.408 Mb indicates association between genotype at this locus and gene expression. No color means there is not an association., whereas blue and red shading indicates whether B6 (*B*) or D2 (*D*) alleles, respectively, are associated with higher *e*xpression. Yellow arrowheads indicate the physical location of *Comt*. Among the BXD family the *D* allele is associated with higher expression of the distal 3′ UTR of *Comt* in all tissues. However, expression of the common coding mRNA probe set is associated with higher expression of the *B* allele in whole brain (WB), cerebellum (CER), hippocampus (HIP) and striatum (STR). In the kidney (KID) and the prefrontal cortex (PFC), expression of coding *Comt* mRNA is also associated with higher expression of the *D* allele. (C) Multiple QTL maps for *Comt* coding exons. Strong *cis-*regulation of expression is observed for all tissues except for the EYE (eye), VTA (ventral tegmental area), and NAC (nucleus accumbens). Purple arrowheads indicate the approximate location of *Comt*. (D) Multiple QTL maps for *Comt* distal 3′ UTR. Significant cis-regulation of *Comt* is observed across all tissues.

We measured expression of *Comt* across multiple tissues in over 100 strains of mice and several large F2 intercrosses using four microrray platforms. Between strains and across tissue we consistently detect large differences in expression for probe sets that target both coding exons and the distal 3′ UTR (Fig. 1A, Figure S1, and Figure S2). We mapped this variation in *Comt* expression in several crosses, including the large BXD family. In this family, *Comt* expression maps to the location of the *Comt* gene itself (Fig. 1C and Fig. 1D). Such differences that map back to the physical location of the gene are referred to as *cis*-regulated QTLs. For example in kidney, variation in the distal 3′ UTR of the gene (probe set 1418701_at) maps to chromosome (Chr) 16 with an extremely high likelihood of the odds (LOD) score of 30 and a 2-LOD support interval from 17.5 to 19.8 Mb (Fig. 1D). This demonstrates that sequence variants in or around the *Comt* gene control its expression. In this case, each copy of the D2 (*D*) allele increases *Comt* mRNA signal by a factor of two (Fig. 1B and www.genenetwork.org).

**Figure 2:**
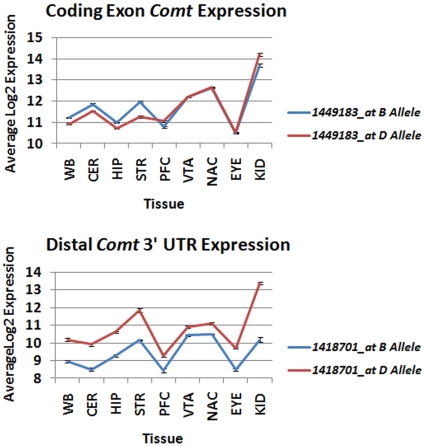
Expression of *Comt* coding exon and distal 3′ UTR in the BXD population across tissues. Probe sets representing either coding exon or distal 3′ UTR were used to measure *Comt* expression in parental and BXD strains. Strain allelic expression for both *Comt* probe sets was determined based on the genotype (B6 or D2) of nearby marker rs4165069 (located on chromosome 16 at 17.577 Mb). Average expression for B6 (*B*) and D2 alleles (*D*) was determined for each probe set across tissues. (Top panel)There is less variation between alleles for *Comt* exon expression across multiple tissues as assayed by probe set 1449183_at. (Bottom Panel) In contrast, there is much greater variation when assayed using the distal 3′UTR (1418701_at). Higher expression of the distal 3′ UTR is consistently associated with the *D* allele while higher expression is associated with the B allele for *Comt* coding exons in the whole brain (WB), cerebellum (CER), hippocampus (HIP), and striatum (STR). Higher expression of coding exons is associated with the D allele in the prefrontal cortex (PFC). Expression of coding exons in the ventral tegmental area (VTA), nucleus accumbens (NAC), eye (EYE), and kidney (KID) is not strongly dependent on genotype.

The expression of mRNA containing the distal 3′ UTR (probe set 1418701_at) is strongly *cis*-modulated in all tissues and is associated with high expression from the *D* allele. In contrast, expression of more proximal coding exons (probe set 1449183_at) is not as strongly *cis*-regulated, is more variable across brain regions and organs, and is usually associated with higher expression from the B6 (*B*) allele (Fig. 1B). Mean expression of the coding exons is higher and less variable among BXD strains than expression of the distal 3′ UTR in all tissues (Fig. 2). For example, there is over 44-fold variation in distal 3′ UTR expression between the *B* and *D* alleles in the kidney compared to a 4-fold difference in coding exons from the same tissue. The pattern of high expression from the *B* allele for more proximal probe sets and high expression from the *D* allele for distal 3′ UTR probe sets was replicated in the hippocampus and striatum using different microarray platforms (Figure S3).

Profound differences in *Comt* expression were detected among highly diverse inbred strains of mice (Figure S1, Figure S2). Like B6, a small number of other common inbred strains, including 129S1/SvImJ, BALB/cByJ, A/J (A), C57BL/6ByJ, and AKR/J (AK), have low expression using the distal 3′ UTR probe set. In contrast, and like D2, a larger number of strains, including C3H/HeJ (H), CBA/CaJ, FVB/NJ, KK/HlJ, NZO/HlLtJ, NZW/LacJ, SJL/J, and all wild subspecies, have high expression. All crosses derived from strains that differed in *Comt* expression show strong *cis*-modulation. For example, progeny of crosses between B6 and H, B6 and CAST/EiJ, and AK and D2 demonstrate *cis*-modulation whereas progeny of crosses between B6 and A, and C57BL/6ByJ and BALB/cByJ do not (Table S1).

**Figure 3:**
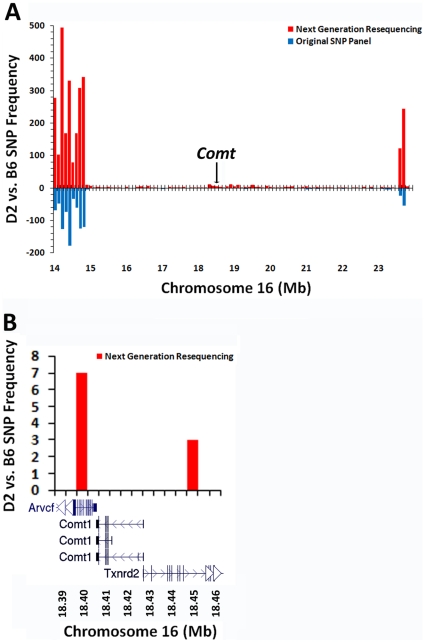
C57BL/6J and DBA/2J are identical by descent for a large region on chromosome 16 surrounding *Comt*. (A) Next-generation sequencing (red) reveals previously unknown SNPs between B6 and D2. The original SNP panel (blue) was obtained at www.ncbi.nlm.nih.gov/projects/SNP/. A large interval surrounding *Comt* from ∼15 to 23 Mb is devoid of SNPs. (B) There are no known SNPs between B6 and D2.

Collectively, these diverse mapping data sets demonstrate that expression of *Comt* is strongly *cis*-regulated and is caused by sequence variation in or around *Comt* itself. Surprisingly, B6, D2, and the great majority of strains are identical by descent for a large interval surrounding *Comt*. There are no known SNPs in *Comt* between B6 and D2 (Fig. 3) and only 212 total SNPs between 15 and 23 Mb. The persistence of strong *cis*-modulation in the absence of SNPs provided a unique opportunity to identify the cryptic, but causal sequence variant in *Comt.*

### Sequencing of the *Comt* locus

**Figure 4:**
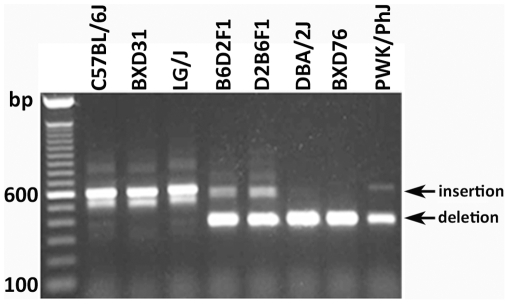
A 230 bp insertion in the proximal 3′ UTR of *Comt* is present in multiple strains. PCR amplification of genomic DNA using primers flanking the 3′UTR (18,407,151 to 18,407,758 Mb) generates two different products. Strains containing the B2 SINE, such as B6, generate a larger PCR product (approximately 600 bp) then do strains lacking the SINE, such as D2 (approximately 400 bp). Genomic DNA from reciprocal hybrids (B6D2F1 and D2B6F1), which are heterozygous for the B2 SINE indel, generates both a short and long product due to inheritance of both B6 and D2 alleles, respectively.

Next-generation whole genome sequencing data has recently been completed for the D2 strain using Applied Biosystems SOLiD and Illumina GA2 sequencing platforms (whole genome shotgun coverage of ∼52x). A complete compendium of new D2 SNPs and other sequence variants in and around *Comt* is available at GeneNetwork (www.genenetwork.org) and from the Wellcome Trust Sanger Institute (http://www.sanger.ac.uk/resources/mouse/genomes/). We identified large numbers of structural variants (copy number variants, translocations, inversions, and insertions and deletions) between B6 and D2, but none in the *Comt* interval. The sole exception was a 230 bp deletion in D2 (Figure S4). This insertion/deletion (indel) was located in the proximal 3′ UTR (Chr 16 from 18,407,621–18,407,392 Mb). We sequenced the region around the indel in 8 strains with high or low expression (Fig. 4) and B6 and D2 reciprocal F1 hybrids. The presence or absence of the indel corresponded to the expression level detected by the distal 3′ UTR probe set (1418701_at). Strains with the insertion, B6, BXD31, LG/J, and A/J (not shown), have low expression whereas strains without the insertion, D2, BXD76, PWK/PhJ, and KK/HIJ (not shown), have high expression.

To rule out the possibility that other sequence variants regulate *Comt* we queried genomic sequence data from seventeen inbred strains available from the Sanger Institute. We found no SNPs, indels, or haplotypes that segregate with the pattern of expression for a ±2 Mb interval surrounding *Comt*. The only exception is an isolated indel that segregates perfectly among all strains with high or low distal 3′ UTR expression. This indel is the only viable candidate for the massive *cis*-QTL in *Comt.* For this reason we closely examined this variant to understand the mechanisms that generate the strong *cis* eQTL.

### Characterization of the 3′ UTR indel in the *comt* locus

**Figure 5:**
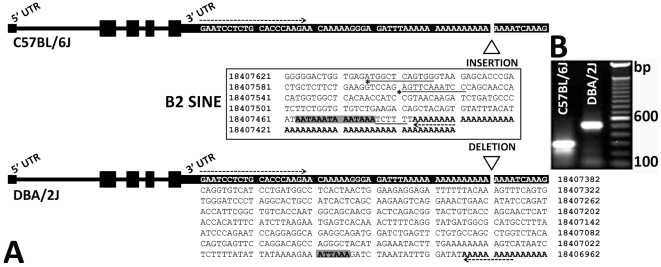
Insertion of a B2 SINE containing an alternative polyadenylation site into the *Comt* gene leads to the production of mRNA containing a shorter 3′ UTR in B6. (A) Gel electrophoresis of 3′ RACE products shows that D2 produces mRNA containing a 3′ UTR that is ∼200 nts longer compared to B6. This is consistent with the truncation of B6 mRNA due to the introduction of the B2 SINE polyadenylation signal (A). Expression data indicate that a small amount of the long 3′ UTR produced in most tissues of strains with the insertion, likely due to read-through of the B2 SINE polyadenylation signal. (B) Black shaded text indicates identical proximal 3′ UTR DNA sequence in B6 and D2 strains. Bold text and grey shaded text indicate the position of a poly (A) tail and polyadenylation signals, respectively. The B2 SINE (sequence in center box) contains both A and B box RNA polymerase III promoters (asterisks and underlined text, respectively), a consensus RNA polymerase III termination signal (underlined text), a polyadenylation signal arranged in an overlapping array (bold text, shaded in grey), and an adenine- (A-) rich 3′ flanking region (bold text). Arrows indicate the approximate position of both a custom *Comt* forward primer (right arrow) and a generic reverse primer (left arrow) used to amplify 3′ RACE products from cDNA for each strain. The exact length of the poly-A tail for each mRNA species from B6 and D2 is not known. Genomic positions based on the B6 reference strain (UCSC Genome Browser on Mouse July 2007 Assembly mm9).

The 230 bp indel is a repetitive B2 family short interspersed element (SINE). B2 SINEs are a common type of retrotransposon representing ∼2.4% of the mouse genome [14]. The B2 sequence contains several typical features, including a split RNA polymerase III promoter and termination signal. Of greater significance, this element also contains an array of overlapping polyadenylation signals flanked by an adenosine (A) rich segment in the 3′ region [15], [16], [17] (Fig. 5A). The insertion of this element and several premature polyadenylation sites in a subset of strains, including B6, should generate mRNA containing a shorter 3′ UTR. In contrast, D2 and other strains that do not contain the SINE, should use the more distal and highly conserved polyadenylation site, and therefore produce a longer 3′ UTR. We used 3′ RACE and RNA sequencing (RNA-seq) to test the effect of the introduced polyadenylation signal on 3′ UTR length. As expected, the B6 strain produced a much shorter 3′ UTR compared to D2 (Fig. 5B) and there was a marked reduction in mRNA containing the distal 3′ UTR in B6 (Figure S5).

The insertion of the B2 SINE produces variation in 3′ UTR length that segregates among BXD strains and is the sole cause of *cis*-regulation of *Comt* expression. In the BXD panel, probe set measurements based on the distal 3′ UTR detect the presence or absence of the long 3′ UTR isoform and show consistent allelic regulation of expression (Figure S1, Figure S2). Probe sets that target coding exons do not discriminate between isoforms and more accurately reflect overall strain and tissue differences in total mRNA level. The genetic control of these probe sets was tissue specific. For example, highest expression of coding exons in the cerebellum, hippocampus, and striatum was associated with the *B* allele, whereas in prefrontal cortex and kidney, higher expression was generally associated with the *D* allele (Fig. 1B and Fig. 2). This demonstrates that transacting factors and tissue specific *trans*-QTLs are also important modulators of *Comt* expression.

**Figure 6:**
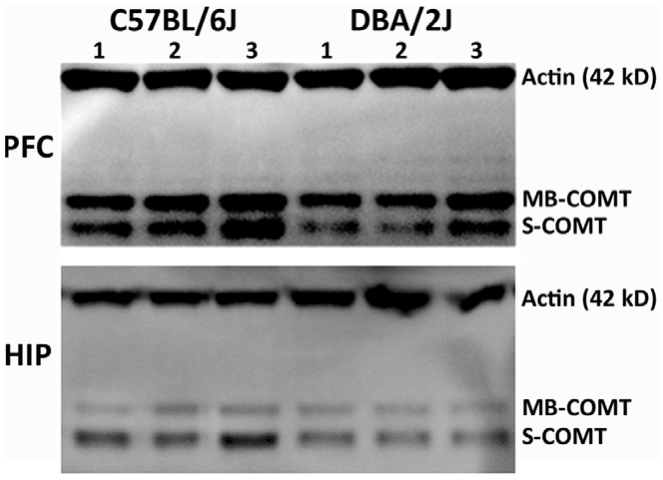
The level of COMT protein differs between the B6 and D2 strains. Lanes 1 through 3 and 4 through 6 represent biological replicates from B6 and D2, respectively. Upper and lower panels show protein levels in the prefrontal cortex and hippocampus, respectively. Actin levels are measured as a loading control. Two protein isoforms of COMT are expressed in mice and recognized by the COMT antibody; the longer membrane bound (MB)-COMT and the smaller soluble (S)-COMT. Both isoforms have higher expression in B6 compared to D2 in the prefrontal cortex (PFC) and hippocampus (HIP). Based on the amount of actin detected in the hippocampus it appears that there may be a slightly higher amount of protein in the D2 samples, however, the expression of MB-COMT and S-COMT is still higher in B6 compared to D2.

Independent of variation in total *Comt* levels, strains with the B2 SINE insertion primarily produce *Comt* with a short 3′ UTR and strains without the insertion produce *Comt* with a long 3′ UTR. It is possible that these differences in 3′ UTR length have a functional consequence on protein level. In rodent brain a single transcript codes for both soluble (S-) and membrane bound (MB-) COMT through a leaky scanning mechanism [18]. We compared the level of both protein isoforms between strains in hippocampus and prefrontal cortex and both are expressed more highly in B6 (Fig. 6). Regardless of relative mRNA abundance between strains, B6 mRNA containing the shorter 3′ UTR is associated with higher levels of COMT protein expression.

### Impact of *Comt* gene sequence variation on downstream biological functions

What sets of higher order phenotypes are downstream of the *Comt* polymorphism? To answer this question we extracted all classical phenotypes in the GeneNetwork BXD phenotype database that map within 2 Mb of *Comt*. The prior probability of mapping near *Comt* is unusually low because this region is small (0.16% of the genome) and, with the exception of the B2 SINE polymorphism, is identical by descent (Fig. 3). Even in massive expression data sets no more than 1–2 transcripts are expected to map to this region by chance. Twelve classical phenotypes with high content validity map to the *Comt* locus (*p*<0.02, Table S2), including binding affinity of both dopamine receptors (DRD1 and DRD2) in caudate, nucleus accumbens, ventral tegmental area, and prefrontal cortex [19]. Linkage of DRD1 and DRD2 binding was sex-specific and only detected in males. Sensitivity to the dopamine receptor antagonist haloperidol [20] and chloradiazepoxide, an allosteric modulator of GABA-A receptors [21], also map to *Comt*.

**Figure 7:**
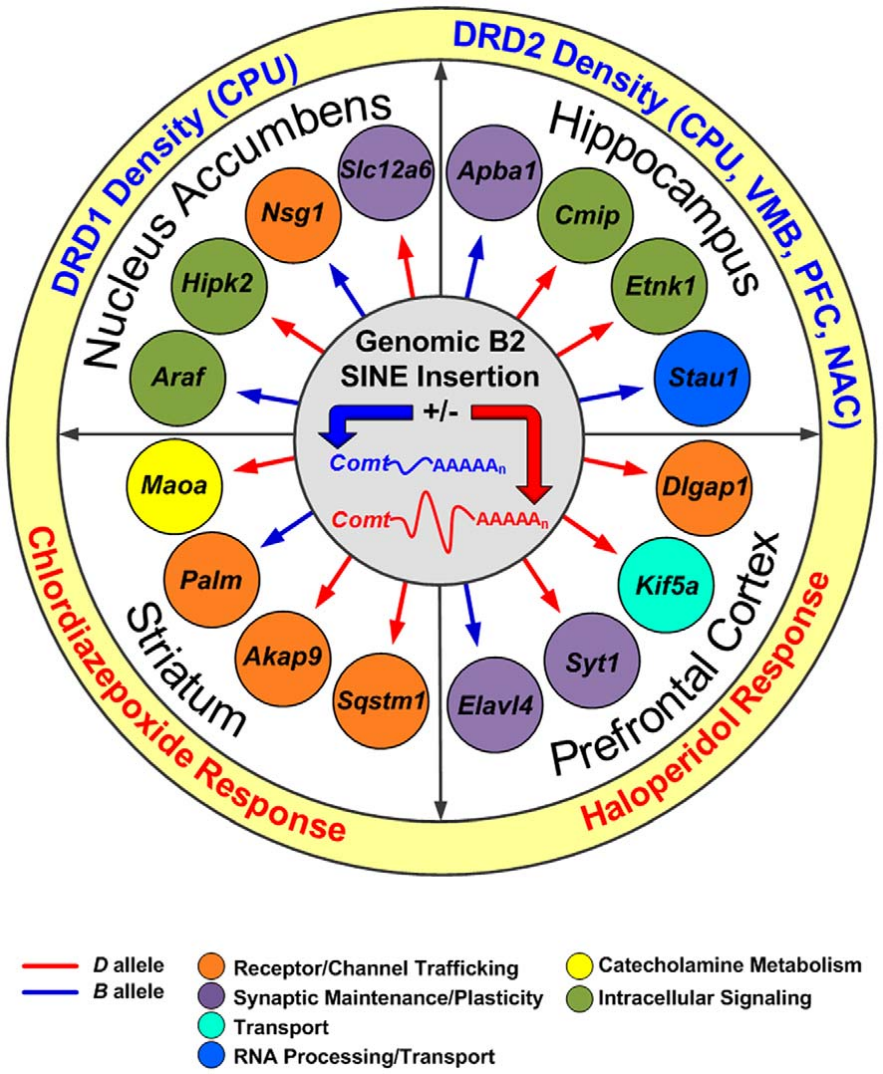
*Comt* regulatory model. Insertion of a B2 SINE causes 3′ UTR length isoforms with important functional consequences. Two concentric rings show the downstream effect *Comt* sequence variation has on the gene expression (white ring) and phenotypes (yellow ring). Variation in *Comt* influences several neurotransmitter systems, including GABA and dopamine, and modulates the expression of genes involved in numerous biological processes, including receptor trafficking at excitatory synapses (*Palm*, *Sqstm1*, *Nsg1, Akap9,* and *Apba1*).

To explore intermediate networks between *Comt* and the classic phenotypes listed above, we extracted transcripts modulated by *Comt* in four brain regions (Fig. 7, Table S3). Transcripts and genes with *trans* QTLs mapping into the *Comt* locus were filtered by expression level, biological relevance, and by examining expression in the Allen Institute for Brain Science (AIBS) brain atlas (www.brain-map.org). Downstream targets of *Comt* participate in cytoskeletal regulation (*Cmip*, *Etnk1*, and *Ptprd*), intracellular signaling (*Cdc42, Araf*, *Hipk2*, *Cmip*, *Etnk1*, *Ptprd,* and *Rasgrp1),* transport (*Stau1*, *Adam10*, *Dnajc10, Mcoln1, Golga3*, *Arl7*, *Spo*, *Pitpnb*, *Ddx47*, *Capns1*, and *Kif5a*), receptor/channel trafficking (*Palm*, *Akap9*, *Sqstm1, Cntn2, Nsg1*, and *Dlgap1*), synaptic maintenance and plasticity (*Apba1*, *Tle3*, *Slit3, Slc12a6, Elavl4*, and *Syt1*), transcriptional regulation (*Nipbl, Myt1l, Ncor1*, *Jundm2*, and *Tef*), and catecholamine metabolism (*Maoa*). Given the marked differences in expression of *Comt* covariates in different tissues and organs, we neither expected nor did we find significant overlap of its downstream targets. One exception was receptor protein tyrosine phosphatase delta (*Ptprd)*. A key signaling molecule in the CNS involved in neurite growth [22], deletion of *Ptprd* leads to learning and feeding impairments, in addition to alterations in hippocampal long-term potentiation [23]. There was significant overlap in gene function across data sets. Well supported categories included receptor trafficking, and synaptic maintenance/plasticity. Each region also included key genes associated with addiction (*Maoa, Ptprd*, and *Slit3*), psychiatric illnesses (*Maoa*, *Myt1l, Slc12a6,* and *Slit3*), and neurodegenerative diseases (*Apba1* and *Slc12a6*).

## Discussion

In this study we have identified the sequence variant underlying a strong *cis*-acting expression quantitative trait locus (eQTL) for the *Comt* gene. *Comt* is located in a large interval that is otherwise devoid of sequence variants between the BXD strains. In combination with strong mapping data, this provides definitive genetic and sequence support that the insertion of the B2 SINE into the proximal 3′ UTR is the ultimate cause of the *Comt* eQTL. Based on expression data and corroborative sequence data, wild derived strains (MOLF/EiJ, CAST/EiJ, PWK/PhJ, and PWD/PhJ) and Swiss mice (FVB/NJ and SJL/J) lack the insertion. Some strains derived from W. E. Castle and C. C. Little's stocks contain the insertion (C57BL/6J, A/J, 129S1/SvlmJ, and BALB/cByJ) whereas others do not (DBA/2J, 129P3/J, CBA/J, and C58/J). In the absence of the B2 SINE, inbred strains produce mRNA with a long 3′ UTR. The insertion introduces a premature polyadenylation site and the production of a short isoform. The propagation of the B2 SINE in several strains derived by Castle and Little, as well as its absence in wild derived strains, is evidence that the long 3′ UTR is ancestral. The short isoform is likely the result of a transposition fixed by inbreeding in the early 1900s. This naturally occurring mutation is likely to be the major source of variation in *Comt* expression among inbred strains.

The B2 SINE is associated with higher expression of *Comt* coding exons in B6 relative to D2 in the cerebellum, hippocampus, and striatum but not in the prefrontal cortex, ventral tegmental area, and nucleus accumbens. The B2 SINE insertion accounts for over 90% of the variance in mRNA length and expression across tissues but it is not the only mechanism governing expression. Approximately 50% of the variance of the common coding form is due to segregating *trans-*effects, non-segregating masking epistatic interactions, and residual error. Intriguingly, our protein analysis reveals that mRNA containing the short 3′ UTR is translated at a higher level than mRNA containing the longer 3′ UTR, regardless of relative isoform abundance in different tissues. This effect may be mediated by differences in secondary structure between isoforms, preferential binding to each isoform by factors regulating transport or translation, or by isoform differences in polyadenylation. The net result however is that the B2 SINE, by causing a change in the length of the 3′ UTR, contributes significantly to genetic variation in COMT protein abundance. An increase in total COMT could be associated with higher enzymatic activity and previous research has demonstrated significantly higher COMT activity in the nucleus accumbens and prefrontal cortex of the B6 strain compared to D2 [10]. These results demonstrate a link between genomic sequence variation, transcript abundance, protein level, and enzymatic activity.

Variation in *Comt* message and protein levels caused by the B2 SINE influences multiple neurotransmitter systems, and has known and novel downstream effects on neuronal signaling and behavior (Fig. 7). Key genes regulated by *Comt* are involved in catecholamine metabolism (*Maoa*), nervous system maintenance (*Slc12a6*), the excitatory postsynaptic density (*Dlgap2*), and receptor trafficking of AMPA (*Palm*, *Sqstm1*, and *Nsg1*) and NMDA (*Akap9* and *Apba1*) receptors. The *Comt* downstream network also modulates synaptic transport (*Stau1*, *Elavl4*, *Kif5a*, and *Syt1*) and intracellular signaling (*Hipk2*, *Araf*, *Cmip*, and *Etnk1*). As expected, dopaminergic systems are clearly downstream of *Comt*, including *Maoa* in the striatum, DRD1 and DRD2 binding density in multiple brain regions, and the response to the dopamine receptor antagonist haloperidol. Finally, *Comt* modulates components of the glutamatergic and GABAergic systems and is linked to the expression of genes involved in excitatory synapse maintenance and locomotor responses to chlordiazepoxide, an allosteric modulator of GABA-A receptors.

The *Comt* variant exposes interconnectivity among neurotransmitter systems. From one perspective this may be surprising given the role of this enzyme in catecholamine catabolism, but even small changes at the end of a biochemical cascade feed back on transmitter synthesis and receptor trafficking to maintain homeostasis. Polymorphisms in *COMT* are often associated with CNS diseases such as schizophrenia, anorexia nervosa, bipolar disorder, anxiety, and substance use disorders [24]. These complex diseases have comparatively high genetic complexity, population heterogeneity, overlapping diagnostic criteria, modest additive effects of single polymorphisms, and a high likelihood that epistatic interactions influence disease risk [25], [26], [27], [28], [29]. The more widespread use of joint studies of mouse and human populations will provide an effective translational bridge. Genes and pathways that we have identified as sensitive to *Comt* variation in mice are attractive candidates in humans. Similarly, genes and pathways identified in human families and genome-wide association studies are attractive candidates to study using mouse reference populations.

There is significant divergence in COMT sequence and activity between mice and humans. A more efficient first initiation site in human COMT mRNA leads to the production of more membrane bound MB-COMT than soluble S-COMT in human brain [30] which, despite having a lower enzyme capacity, has a higher affinity for dopamine and other neurotransmitters [31], [32], [33]. In contrast, mice express both protein isoforms abundantly in brain. In humans multiple SNPs have been identified that lead to changes in mRNA, protein, and enzyme activity. In contrast, common strains of mice have little variation in *Comt* sequence, the B2 SINE, excepted. Additionally, mouse COMT, which contains Leu instead of Val at the equivalent position as the human Val108/158Met polymorphism, has higher activity than the Val allele [8]. In fact, amino acid substitution from Leu in mice to Val in monkeys and Met in humans substantially reduces COMT activity. This results in increased brain dopamine signaling in humans with important cognitive consequences. For example, Met polymorphisms, which decrease COMT activity, enhance working memory and attention [5], [34], [35], [36], [37], [38], [39]. However, the Met allele is associated with less pain and stress resiliency compared to the Val allele [40]. Despite the differences in the genetic regulation of *Comt* between mouse and human, manipulations point to very similar roles in cognition and behavior. Consistent with human studies, disruption of *Comt* in male mice elevates dopamine levels in the prefrontal cortex [2] and enhances working memory [30]. Conversely, overexpression of human COMT containing the Val polymorphism in mice leads to the disruption of working memory and attention and a reduction in sensitivity to pain and stress [30].

Mammalian genomes contain a large proportion of transposable elements (TEs), including the Alu family of SINEs in humans, and B1 and B2 SINEs in rodents. TE insertions, such as the B2 SINE in *Comt*, are important drivers of gene evolution [16], [41]. Approximately 7% of genes in mice contain non-conserved polyadenylation sites derived from TEs [41]. These polyadenlyation sites can be co-opted to create novel mRNA structures that effect message processing, distribution, and translation. As in the case of *Comt*, insertion of a B2 SINE in the 3′ UTR of major histocompatibility complex genes results in preferential usage of a premature polyadenylation signal [15]. Given the abundance of B1 and B2 SINES—564,000 and 348,000 copies, respectively [14]—it is highly likely that insertions of TEs modulate the expression of several other genes. These types of mutations, combined with intense selection, may have contributed to the rapid behavioral evolution of both wild populations and laboratory strains [42].

## Methods

### Ethics statement

All animal work was conducted according to an approved animal use protocol (UTHSC680) and in accordance with procedures approved by the Institutional Animal Care and Use Committee.

### Expression and QTL analysis of *Comt* using GeneNetwork

Multiple expression datasets from many brain and peripheral tissues of inbred and RI strains were used in this analysis (see Text S1 for additional methods). Additional detailed descriptions of strain, sex, tissue preparation and microarray method for each individual database is available at www.genenetwork.org. Four microarray platforms were used in this analysis including; Agilent, Illumina, Affymetrix Exon 1.0 ST, and M430 arrays. Tools for QTL mapping and visualization are available at www.genenetwork.org [43], [44].

### Comt 3′ UTR genomic DNA isolation and sequencing

Mice were purchased from Jackson Laboratory or bred in-house. Mice were housed at the University of Tennessee Health Science Center in a pathogen-free colony in accordance with procedures approved by the Institutional Animal Care and Use Committee. Naive B6, D2, BXD31, BXD76, B6D2F1, D2B6F1, A/J, LG/J, PWK/PhJ, and KK/HlJ mice were killed at about 60 days of age by cervical dislocation.The liver was immediately collected and stored in RNALater (Ambion) at 4°C overnight. Genomic DNA was isolated using DNeasy Kits (QIAGEN) We used Primer 3 software to design custom forward (5′-GCG CCA TCA TAC CTG AAA AG-3′) and reverse (5′-GGA AAA CAC CTG TGC ATCA A-3′) primers specifically targeting *Comt* 3′ UTR genomic DNA for amplification by PCR. PCR product size was predicted to be 608 bp based on available genomic data for the B6 reference strain. Coverage area within the *Comt* locus was from 18,407,151 to 18,407,758 Mb. DNA amplification was performed using the HotStar HiFidelity Polymerase kit (Qiagen) and 10 ng of genomic DNA for each strain followed by sequencing of the PCR products with each custom primer using an A3130XL Genetic Analyzer (Applied Biosystems). The resulting sequences were aligned against reference sequences from the NCBI GenBank database using nucleotide blast. The sequences were also aligned between strains using BLAST (bl2seq). The genomic DNA sequence of the *Comt* 3′ UTR for the D2 strain is available at GenBank (GU324996).

### Expression analysis using RNA -seq

Total RNA was isolated from the whole brains of 27BXD RI strains using RNA STAT-60 (Tel-Test Inc). Samples were DNase treated using a DNA-free kit (Ambion) to eliminate genomic DNA. The quality and purity of total RNA from each sample was assessed using an Agilent Bioanalyzer 2100 system. For RNA-seq, ribosomal RNA (rRNA) was removed from each total RNA sample using the RiboMinus Eukaryote Kit (Invitrogen). Each RNA sample was then converted to cDNA and amplified. Emulsion PCR was performed for each sample and each fragment library was subsequently sequenced on the Applied Biosystems SOLiD platform. This procedure preserves the strandedness (plus or minus) of the RNA read.

### RNA-seq data processing and visualization

Short sequence reads were analyzed using Applied Biosystems whole transcriptome software tools (www.solidsoftwaretools.com/).Reads were mapped to the B6 reference genome (mm9, US National Center for Biotechnology Information (NCBI) build 37) with a minimum alignment score of 24.

### Isolation and sequencing of *Comt* 3′ UTR mRNA

RNA STAT-60 (Tel-Test Inc) was used for RNA extraction from B6 and D2 hippocampus followed by ethanol precipitation. RNA quality was determined using a NanoDrop spectrophotometer (Thermo Scientific) and Agilent Bioanalyzer 2100 (Agilent). Target cDNA was generated using the FirstChoice RLM-RACE kit (Ambion) with minor modifications to the standard protocol (Text S1). The amplified products for each strain were purified with a PCR purification kit (Qiagen) and sequenced using both *Comt* 3′ UTR forward primer and the 3′ RACE inner primer separately. The mRNA sequence data for the B6 and D2 strain is available at GenBank (GU324998 contains B6 3′ UTR mRNA sequence and GU324997 contains D2 3′ UTR mRNA sequence). The full length of the 3′ UTR is 553 and 789 nt for the B6 and D2 strain, respectively.

### Protein extraction and immunoblotting

Fresh brain tissue from the hippocampus and prefrontal cortex was dissected from B6 and D2, snap-frozen in liquid nitrogen, and stored at −80°C followed by protein extraction (Text S1). Aliquots were created from each total protein extract and stored at −80°C until used for immunoblotting. Six total protein samples each from the hippocampus and prefrontal cortex of the B6 and D2 strain were used. Fifteen ug of protein was loaded on a NuPAGE 4−12% Bis-Tris Gel (Invitrogen). Protein samples were separated by electrophoresis at 150 volts for 45 min before transferring to a PVDF membrane. Immunoblotting was performed using the WesternDot 625 goat anti-mouse Western blot kit (Invitrogen) according to the manufacturer's instructions (Text S1). Primary antibodies used in immunoblotting were mouse anti-COMT (clone 4/COMT, aa 26–141) and mouse anti-actin Ab5 (BD Transduction Laboratories). Images were acquired with an AlphaImager HP (Alpha Innotec).

### Mapping traits to the *Comt* locus

We used a point-wise *p*-value of <0.02 (LOD>1.73) to identify traits downstream of *Comt*. Phenotypes within the BXD Phenotype database were correlated with marker rs4165069 followed by marker regression to select traits with a significant marker association. Probe sets mapping to *Comt* were identified using the advanced search option in GeneNetwork across several Affymetrix M430 and Exon brain databases (Text S1). The advanced search option was used to detect probe sets with at least an LRS of 8 (LOD>1.73; *p*<0.02) and a mean expression level of 8 and 11 in the M430 and exon databases, respectively. False discovery rates for eQTL were conditioned by the likelihood that traits would map near *Comt* based on a target interval of 4 Mb and the relative density of sequence variants that produce eQTLs. Given these priors we expected less than 2 *trans* eQTL would map to the *Comt* interval by chance. Out of 24, 60, 18, and 30 probe sets mapping to the *Comt* locus in the hippocampus, striatum, prefrontal cortex and nucleus accumbens, respectively, we used our filtering criteria to select the 10 strongest traits controlled by variation in *Comt* from each region. Top probe sets were selected based on probe set specificity, strength of association to the *Comt* locus, biological function, and expression pattern for each relevant brain region in the AIBS (www.brain-map.org).

## Supporting Information

Figure S1 Strain variation in expression of distal 3′UTR *Comt* mRNA in whole brain and hippocampus. Mean log 2 expression values depicted above on the y-axis are from the UCHSC BXD Whole Brain M430 2.0 (Nov06) RMA database and the Hippocampus Consortium M430v2 (Jun06) RMA. Strains are indicated by the x-axis. Average expression across databases is 8 log2 units. Arrows indicate parental strains and numbers indicate recombinant inbred line (e.g. 40 = BXD40). (A) Both BXD and other inbred strains separate into two expression groups with either B6-like low expression or D2-like high expression of *Comt* mRNA containing distal 3′UTR sequence in the whole brain. (B) The same pattern of expression is observed in the hippocampus for BXD and other inbred strains. BXD and CXB parental strains are designated by the red and black arrows, respectively. When strains that share the same distal 3′ UTR mRNA expression pattern are crossed, as in the CXB strains (C57BL/6ByJ x BALB/cByJ), no expression differences are observed.
(0.56 MB TIF)

Figure S2 Strain variation in expression of coding exon *Comt* mRNA (1449183_at) in whole brain and hippocampus. Mean log 2 strain expression values are shown on the y-axis from the (A) UCHSC BXD Whole Brain M430 2.0 (Nov06) RMA database and the (B) Hippocampus Consortium M430v2 (Jun06) RMA. Strains are identified on the x-axis. Average expression across databases is 8. Individual BXD lines are identified by a number only (e.g., 16 = BXD16). There is a wide range of strain variation in the expression of coding exon mRNA which detects all expressed isoforms of *Comt* mRNA. Although there are high and low expressing strains, segregation into two distinct expression groups is not as readily apparent as was observed for the expression of the distal 3′ UTR. Black and red arrows indicate parental strains for the BXD and CXB strains (C57BL/6By x BALB/cBy), respectively.
(0.68 MB TIF)

Figure S3 Replication of *Comt* expression and genetic regulation in the hippocampus using Affymetrix Exon 1.0 ST arrays. (A) Location of probe sets to the distal 3′ UTR (4989545) and the last exon (5131400) of *Comt* based on BLAT search in the UCSC Genome Browser Mouse July 2007 Assembly (mm9). (B) Both probe sets are strongly *cis*-regulated from a region on chromosome 16 near the physical location of the *Comt* gene. (C) The B allele drives expression of the last exon while the D allele drives expression of the distal 3′ UTR.
(0.28 MB TIF)

Figure S4 Sequence alignment of C57BL/6J (B6) and DBA/2J (D2) genomic DNA in the chromosome 16 region containing the indel. There is a 230 bp insertion in the B6 strain, absent in the D2 strain. Red and black indicate genomic sequence from the D2 and B6, respectively. The corresponding genomic position is based on the (-) strand for the B6 reference strain. Dashes indicate the indel position.
(0.24 MB TIF)

Figure S5 Validation of the effect of the B2 SINE insertion on distal 3′ UTR expression by RNA-seq. RNA sequencing was performed on whole brain samples from 27 BXD strains as described in the methods. Strains were subdivided into a B or D allele group for the *Comt* locus based on genotype at marker rs4165081. The B allele group included 10 strains and the D allele group included17 strains. The *Comt* locus was subdivided into six features and the total number of 50-nt sequence tags mapping to each feature was determined for all strains. Tag counts were normalized by dividing by the total number per million in each sample. Normalized tag counts are shown for the B and D allele for each feature. A t-test revealed a significant difference between the B and D allele for the distal 3′ UTR (p = 0.015; **) and the second to last coding exon (p = 0.044; *). No sequence tags map exclusively to the B2 SINE because it is a repetitive element and similar sequences are scattered throughout the genome.
(0.13 MB TIF)

Table S1 Summary of *Comt* regulation.
(0.03 MB DOC)

Table S2 Published phenotypes mapping to the *Comt* locus.
(0.04 MB DOC)

Table S3 BXD exon and M430 top probe sets mapping to the *Comt* locus.
(0.26 MB DOC)

Text S1 Supplemental methods.
(0.07 MB DOC)
