# Supplementary figures and images for: A Transposon in Comt Generates mRNA Variants and Causes Widespread Expression and Behavioral Differences among Mice
Source: PLoS One. 2010 Aug 17;5(8):e12181. doi: 10.1371/journal.pone.0012181 (PMC2923157; doi:10.1371/journal.pone.0012181)

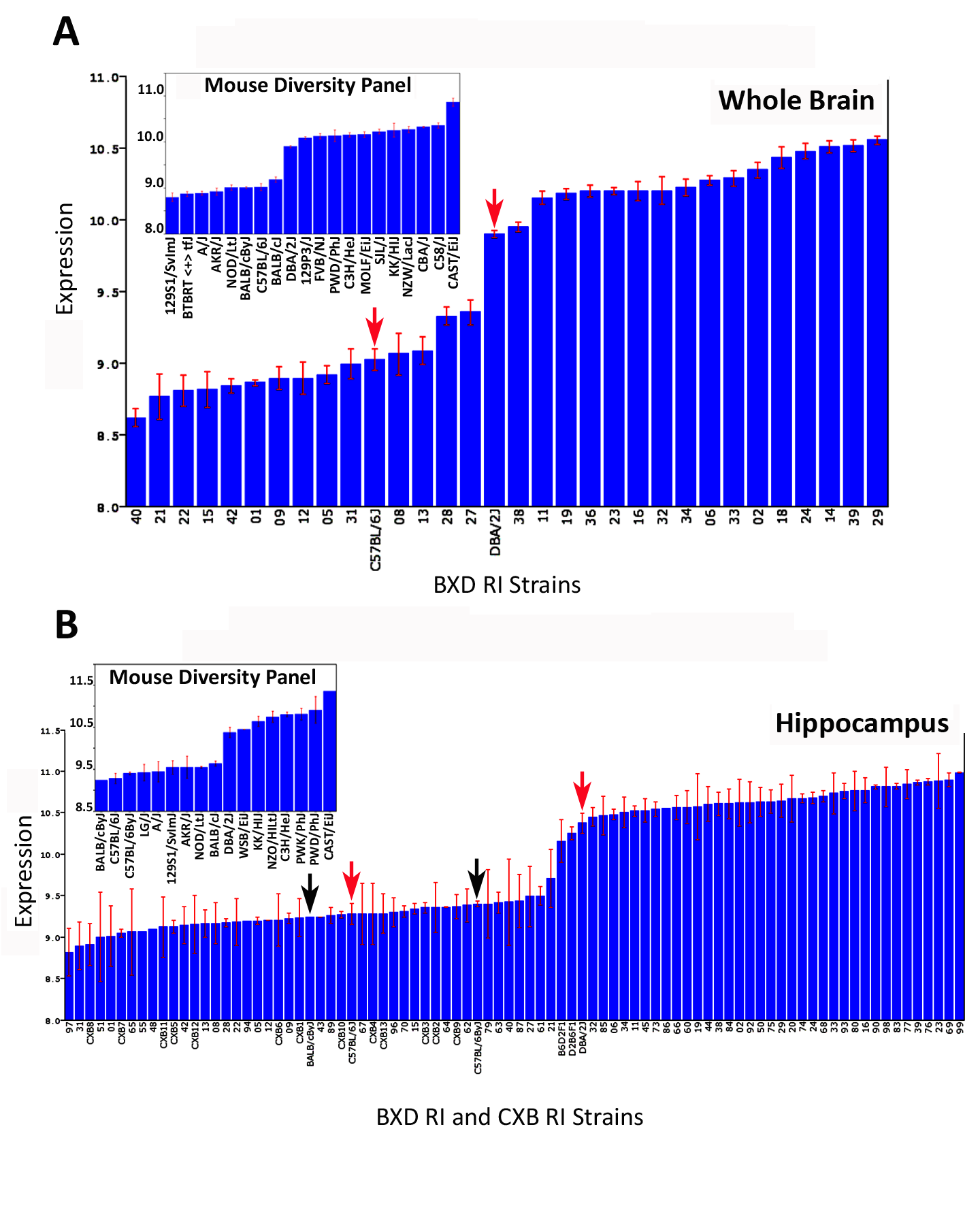

Supplement: Figure S1 — Strain variation in expression of distal 3′UTR Comt mRNA in whole brain and hippocampus. Mean log 2 expression values depicted above on the y-axis are from the UCHSC BXD Whole Brain M430 2.0 (Nov06) RMA database and the Hippocampus Consortium M430v2 (Jun06) RMA. Strains are indicated by the x-axis. Average expression across databases is 8 log2 units. Arrows indicate parental strains and numbers indicate recombinant inbred line (e.g. 40 = BXD40). (A) Both BXD and other inbred strains separate into two expression groups with either B6-like low expression or D2-like high expression of Comt mRNA containing distal 3′UTR sequence in the whole brain. (B) The same pattern of expression is observed in the hippocampus for BXD and other inbred strains. BXD and CXB parental strains are designated by the red and black arrows, respectively. When strains that share the same distal 3′ UTR mRNA expression pattern are crossed, as in the CXB strains (C57BL/6ByJ x BALB/cByJ), no expression differences are observed. (0.56 MB TIF) [file pone.0012181.s001.tif]

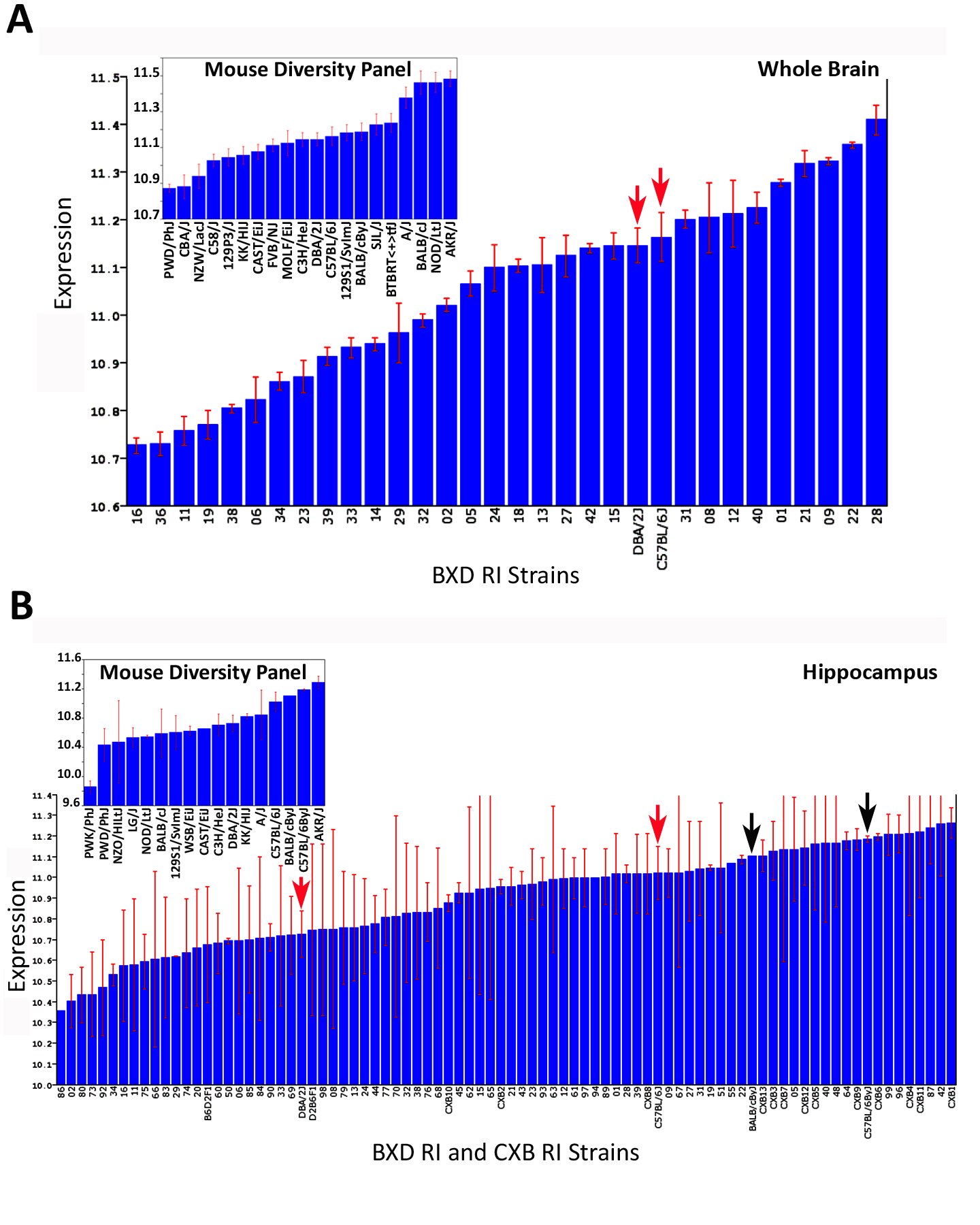

Supplement: Figure S2 — Strain variation in expression of coding exon Comt mRNA (1449183_at) in whole brain and hippocampus. Mean log 2 strain expression values are shown on the y-axis from the (A) UCHSC BXD Whole Brain M430 2.0 (Nov06) RMA database and the (B) Hippocampus Consortium M430v2 (Jun06) RMA. Strains are identified on the x-axis. Average expression across databases is 8. Individual BXD lines are identified by a number only (e.g., 16 = BXD16). There is a wide range of strain variation in the expression of coding exon mRNA which detects all expressed isoforms of Comt mRNA. Although there are high and low expressing strains, segregation into two distinct expression groups is not as readily apparent as was observed for the expression of the distal 3′ UTR. Black and red arrows indicate parental strains for the BXD and CXB strains (C57BL/6By x BALB/cBy), respectively. (0.68 MB TIF) [file pone.0012181.s002.tif]

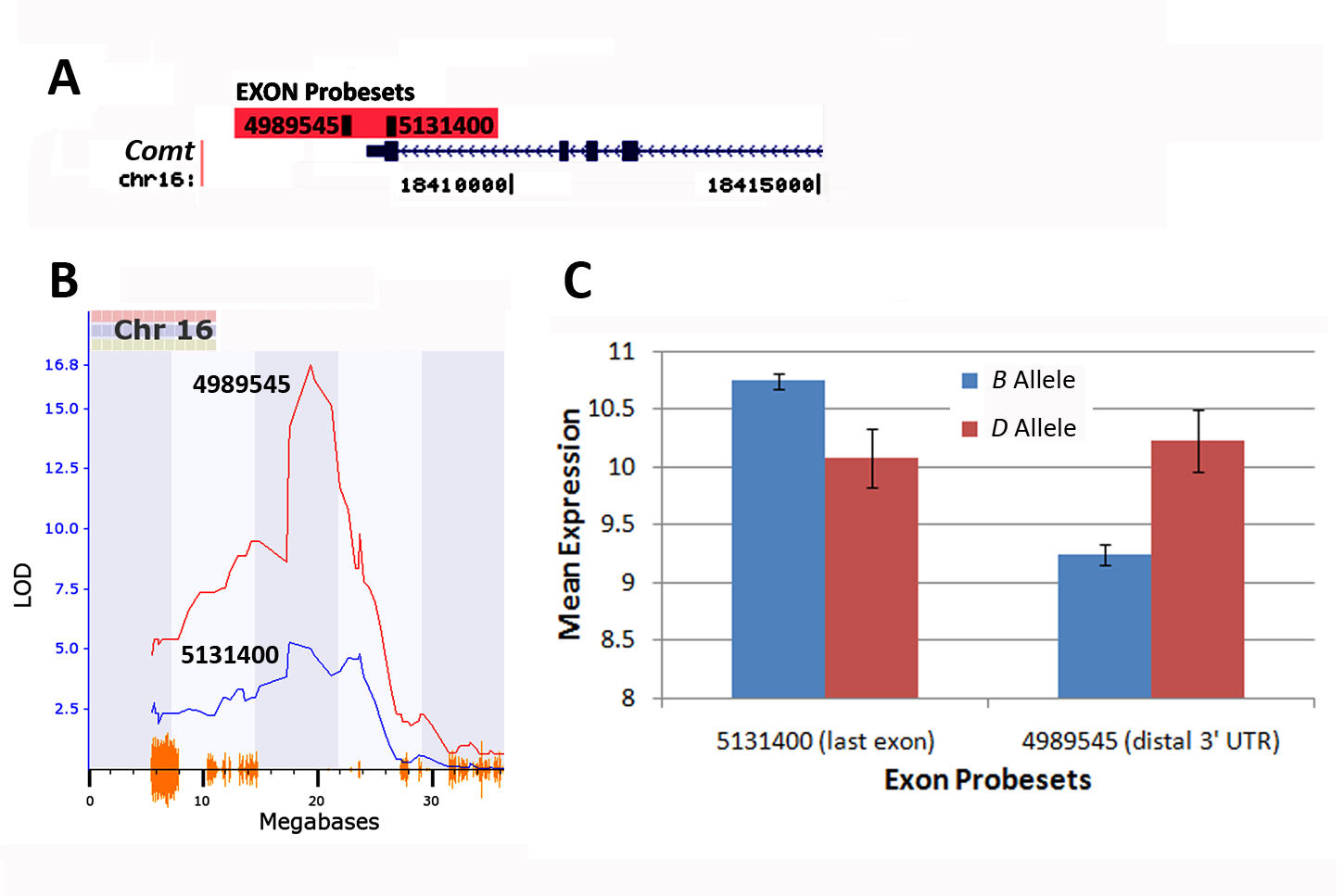

Supplement: Figure S3 — Replication of Comt expression and genetic regulation in the hippocampus using Affymetrix Exon 1.0 ST arrays. (A) Location of probe sets to the distal 3′ UTR (4989545) and the last exon (5131400) of Comt based on BLAT search in the UCSC Genome Browser Mouse July 2007 Assembly (mm9). (B) Both probe sets are strongly cis-regulated from a region on chromosome 16 near the physical location of the Comt gene. (C) The B allele drives expression of the last exon while the D allele drives expression of the distal 3′ UTR. (0.28 MB TIF) [file pone.0012181.s003.tif]

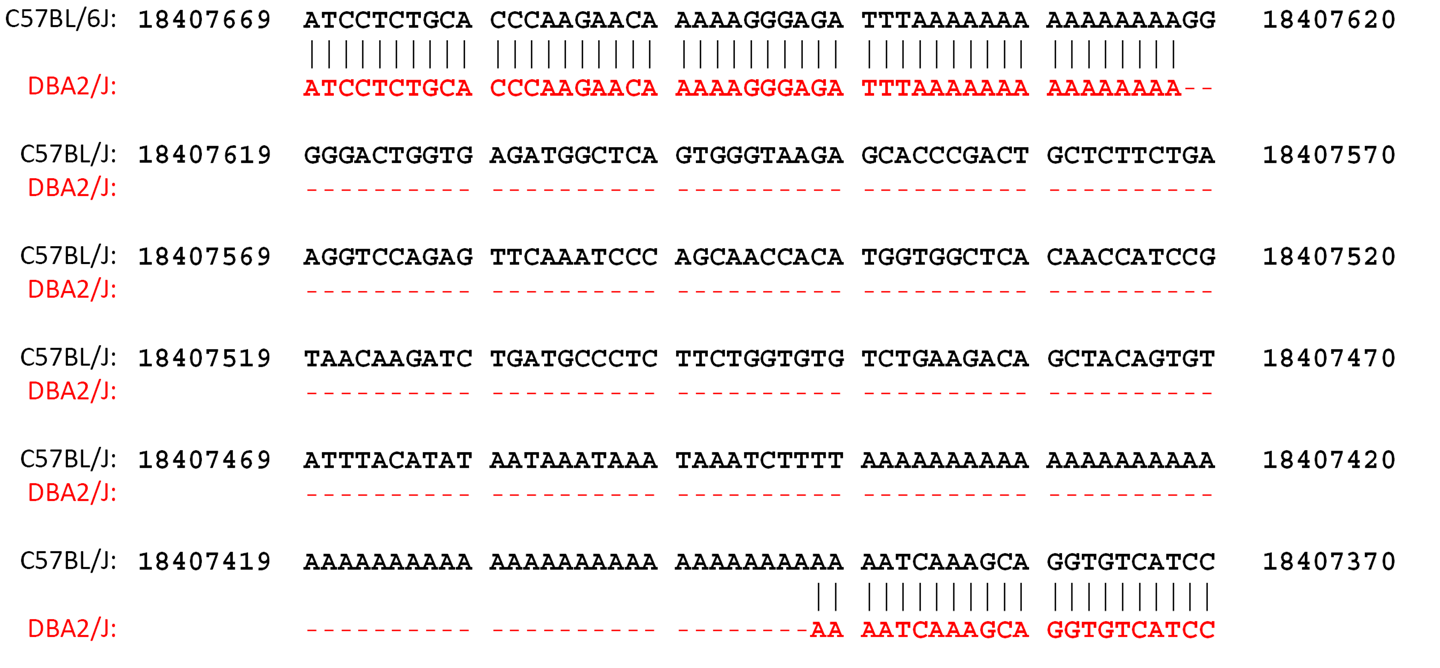

Supplement: Figure S4 — Sequence alignment of C57BL/6J (B6) and DBA/2J (D2) genomic DNA in the chromosome 16 region containing the indel. There is a 230 bp insertion in the B6 strain, absent in the D2 strain. Red and black indicate genomic sequence from the D2 and B6, respectively. The corresponding genomic position is based on the (-) strand for the B6 reference strain. Dashes indicate the indel position. (0.24 MB TIF) [file pone.0012181.s004.tif]

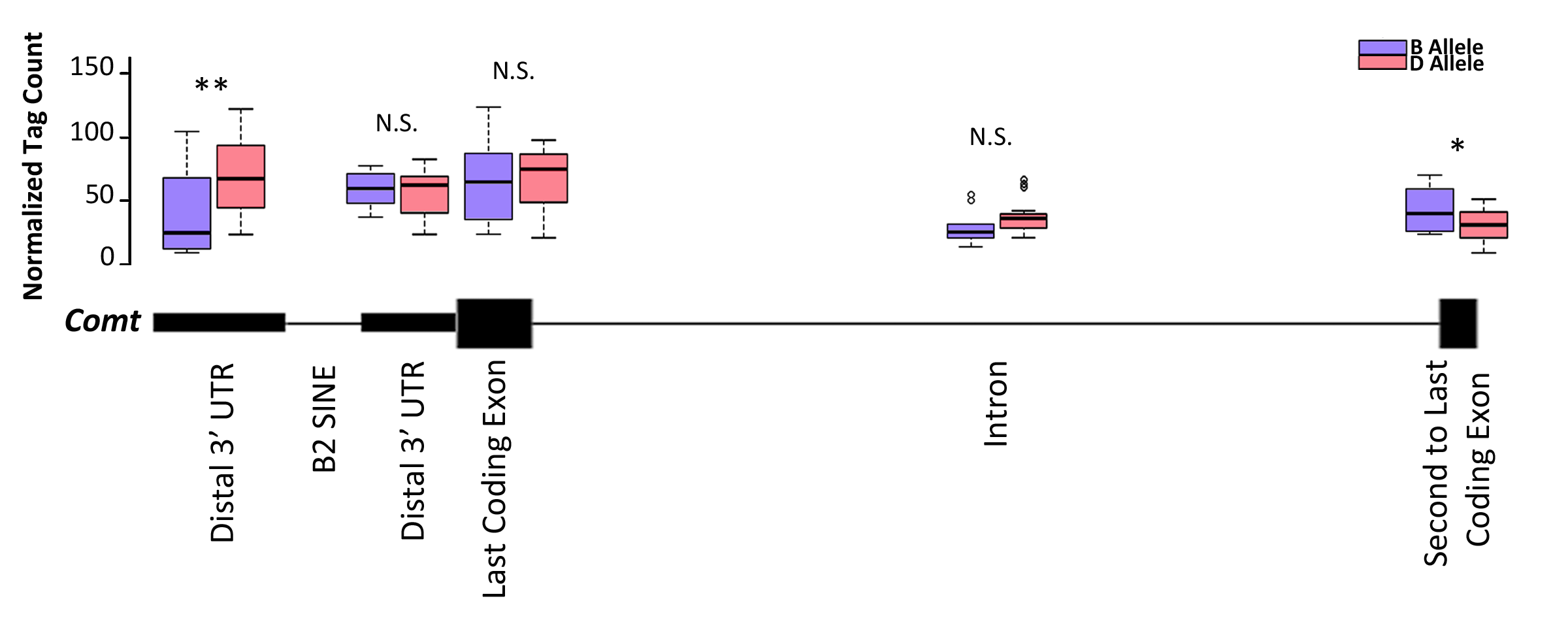

Supplement: Figure S5 — Validation of the effect of the B2 SINE insertion on distal 3′ UTR expression by RNA-seq. RNA sequencing was performed on whole brain samples from 27 BXD strains as described in the methods. Strains were subdivided into a B or D allele group for the Comt locus based on genotype at marker rs4165081. The B allele group included 10 strains and the D allele group included17 strains. The Comt locus was subdivided into six features and the total number of 50-nt sequence tags mapping to each feature was determined for all strains. Tag counts were normalized by dividing by the total number per million in each sample. Normalized tag counts are shown for the B and D allele for each feature. A t-test revealed a significant difference between the B and D allele for the distal 3′ UTR (p = 0.015; **) and the second to last coding exon (p = 0.044; *). No sequence tags map exclusively to the B2 SINE because it is a repetitive element and similar sequences are scattered throughout the genome. (0.13 MB TIF) [file pone.0012181.s005.tif]
