# Supplementary material for: A Transposon in Comt Generates mRNA Variants and Causes Widespread Expression and Behavioral Differences among Mice
Source: PLoS One. 2010 Aug 17;5(8):e12181. doi: 10.1371/journal.pone.0012181 (PMC2923157; doi:10.1371/journal.pone.0012181)
Supplement: Table S1 — Summary of Comt regulation. (0.03 MB DOC) [file pone.0012181.s006.doc]

Table S1. Summary of *Comt* Regulation

| **Database** | **Cross** | **Tissue** | ***Comt* Probe Set ID** | **Marker** | **LOD** | **LRS** | **cis-regulation** |
| --- | --- | --- | --- | --- | --- | --- | --- |
| UCLA BHF2 Brain (June05) mlratio | C57BL/6J  x  C3H/HeJ | Brain | 10024399792 | rs3714738 | 2.48 | 11.42 | Suggestive |
| NCI Mammary mRNA M430 (July04) RMA | (AKR/J  x  DBA2/J F1) x  FVB/NJ | Mammary Tumors | 1418701_at_A | rs4165081 | 4.31 | 19.89 | Significant |
| UCLA CTB6/B6CTF2 Liver (2005) mlratio | C57BL/6J  x  CAST/EiJ F2 | Liver | 10019061620 | mCV24708470 | 35.23 | 162.39 | Significant |
| Eye AXBXA Illumina V6.2(Oct08) RankInv Beta | C57BL/6J  x  A/J | Eye | ILMN_1248478 | rs4165069 | 0.31 | 1.45 | Not significant |
| Hippocampus Consortium M430v2 CXB (Dec05) RMA | C57BL/6By x  BALB/cBy | Hippocampus | 1418701_at | rs4165069 | 0.11 | 0.50 | Not significant |
| **LOD=Likelihood of the odds ratio; LRS=Likelihood ratio statistic** | | | |  |  |  |  |
