# Supplementary material for: A Transposon in Comt Generates mRNA Variants and Causes Widespread Expression and Behavioral Differences among Mice
Source: PLoS One. 2010 Aug 17;5(8):e12181. doi: 10.1371/journal.pone.0012181 (PMC2923157; doi:10.1371/journal.pone.0012181)
Supplement: Table S2 — Published phenotypes mapping to the Comt locus. (0.04 MB DOC) [file pone.0012181.s007.doc]

| **GN ID** | **Allele** | **PubMed ID** | **Phenotype** | **Marker LRS** | **Marker LOD** | **Marker Add** | **Marker Corr** | **N** | **p-value** | **Mean Expression** |
| --- | --- | --- | --- | --- | --- | --- | --- | --- | --- | --- |
| 10264 | *B* | 10591541 | Dopamine receptor 2 (DRD2) protein density in dorsal striatum (caudate putamen) of males [fM Schering Compound 3H-23390] | 13.41 | 2.91 | -83.18 | -0.72 | 18 | 3.79E-04 | 219.78 |
| 10272 | *B* | 10591541 | Dopamine receptor 2 (DRD2) protein density in the ventral midbrain (including the ventral tegmental area and substantia nigra) of males and females [fM Schering Compound 3H-23390] | 11.98 | 2.60 | -8.79 | -0.70 | 18 | 8.40E-04 | 47.83 |
| 10252 | *B* | 10591541 | Dopamine receptor 1 (DRD1) protein density in dorsal striatum (caudate putamen) of males [fM Schering Compund 3H-23390] | 11.83 | 2.57 | -403.89 | -0.71 | 17 | 9.52E-04 | 1530.13 |
| 10904 | *B* | 18185497 | Anxiety assay, locomotion in the light compartment relative to total in a light-dark test [%] | 11.62 | 2.52 | -4.06 | -0.73 | 15 | 1.16E-03 | 31.88 |
| 10267 | *B* | 10591541 | Dopamine receptor 2 (DRD2) protein density in the prefrontal cortex of males [fM Schering Compound 3H-23390] | 10.05 | 2.18 | -10.14 | -0.65 | 18 | 2.44E-03 | 49.57 |
| 10270 | *B* | 10591541 | Dopamine receptor 2 (DRD2) protein density in ventral midbrain (including the ventral tegmental area and substantia nigra) of males [fM Schering Compound 3H-23390] | 8.94 | 1.94 | -9.90 | -0.63 | 18 | 4.47E-03 | 46.33 |
| 10261 | *B* | 10591541 | Dopamine receptor 2 (DRD2) protein density in the nucleus accumbens of males [fM Schering Compound 3H-23390] | 8.72 | 1.89 | -35.33 | -0.62 | 18 | 5.02E-03 | 181.46 |
| 10064 | *B* | 9655868 | Area under the 25-hr curve for withdrawal following 72 hr exposure to air. Pyrazole injections were given daily to inhibit EtOH metabolism. Handling-induced convulsions (HIC) were scored hourly for 10 hr and again at hrs 24 and 25 [seizure severity] | 8.59 | 1.86 | -7.08 | -0.54 | 25 | 4.67E-03 | 11.95 |
| 10123 | *D* | 9880575 | Chlordiazepoxide response (10 mg/kg ip), locomotion from 15-20 min after injection [cm] | 11.34 | 2.46 | 447.03 | 0.60 | 25 | 1.04E-03 | -202.56 |
| 10336 | *D* | 8627512 | Haloperidol response, induced catalepsy at ED50 [mg/kg] | 10.49 | 2.27 | 1.08 | 0.58 | 26 | 1.64E-03 | 2.62 |
| 10124 | *D* | 9880575 | Chlordiazepoxide 10 mg/kg induced locomotor response distance traveled, 5-20 min time interval [cm] | 8.58 | 1.86 | 1155.16 | 0.54 | 25 | 4.71E-03 | -193.67 |
| 12546 | *D* | Unpublished | Transferrin saturation of 120-day-old females fed 270 ppm iron diet [%] | 8.20 | 1.78 | 4.21 | 0.56 | 22 | 6.07E-03 | 31.03 |

Table S2. BXD Published Phenotypes Mapping to the *Comt* Locus

**GN ID=GeneNetwork ID; Marker LRS= Likelihood Ratio Statistic; Marker LOD=Likelihood of the Odds Ratio; Marker Add=Additive Effect; Marker Corr = Correlation (Pearson's *r*)**
