# Supplementary material for: A Transposon in Comt Generates mRNA Variants and Causes Widespread Expression and Behavioral Differences among Mice
Source: PLoS One. 2010 Aug 17;5(8):e12181. doi: 10.1371/journal.pone.0012181 (PMC2923157; doi:10.1371/journal.pone.0012181)
Supplement: Table S3 — BXD exon and M430 top probe sets mapping to the Comt locus. (0.26 MB DOC) [file pone.0012181.s008.doc]

**Table S3. BXD Exon and M430 Top Probe Sets Mapping to the *Comt* Locus.**

| **Database** | **Probe Set** | **Symbol** | **Description** | **Function** | **Ref** | **Chr** | **Mb** | ***Comt* Marker** | **LOD** | **Mean Expr** | **Allele** |
| --- | --- | --- | --- | --- | --- | --- | --- | --- | --- | --- | --- |
| Hippocampus Consortium M430v2 (Jun06) RMA | 1453015_at | Cmip | c-Maf-inducing protein C-mip | CR; ISC | [1] | 8 | 119.98 | rs4165069 | 2.61 | 12.89 | *D* |
| UMUTAffy Hippocampus Exon (Feb09) RMA | 5399675 | Ptprd | protein tyrosine phosphatase, receptor type, D | CR; ISC; APDA | [2] | 4 | 75.73 | rs4165065 | 1.99 | 12.66 | *D* |
| UMUTAffy Hippocampus Exon (Feb09) RMA | 5176789 | Etnk1 | ethanolamine kinase 1 | ISC | [3] | 6 | 143.14 | rs4165081 | 2.53 | 12.86 | *D* |
| Hippocampus Consortium M430v2 (Jun06) RMA | 1450741_at | Stau1 | staufen 1 | RT | [4,5] | 2 | 166.77 | rs4165065 | 2.48 | 9.41 | *B* |
| UMUTAffy Hippocampus Exon (Feb09) RMA | 5157813 | Stau1 | staufen 1 | RT | [4,5] | 2 | 166.78 | rs4165081 | 2.89 | 11.85 | *B* |
| UMUTAffy Hippocampus Exon (Feb09) RMA | 4979476 | Adam10 | a disintegrin and metallopeptidase domain 10 | PT; ISC; APDA |  | 9 | 70.63 | rs4165081 | 2.37 | 11.03 | *D* |
| UMUTAffy Hippocampus Exon (Feb09) RMA | 4432094 | Dnajc10 | DnaJ (Hsp40) homolog, subfamily C, member 10 | PT |  | 2 | 80.19 | rs4165069 | 2.99 | 11.99 | *D* |
| Hippocampus Consortium M430v2 (Jun06) RMA | 1459605_at | Apba1 | amyloid beta (A4) precursor protein binding, family A, member 1 | SMP; APDA | [6,7] | 19 | 23.97 | rs4165069 | 2.63 | 8.33 | *B* |
| UMUTAffy Hippocampus Exon (Feb09) RMA | 5348903 | Tle3 | transducin-like enhancer of split 3 | SMP |  | 9 | 61.26 | rs4165081 | 3.37 | 11.05 | *B* |
| UMUTAffy Hippocampus Exon (Feb09) RMA | 5047183 | Slit3 | slit homolog 3 | SMP; APDA | [8,9] | 11 | 35.45 | rs4165069 | 1.88 | 11.35 | *B* |
| UMUTAffy Hippocampus Exon (Feb09) RMA | 5440667 | Nipbl | Nipped-B homolog | TR; APDA |  | 15 | 8.32 | rs4165065 | 2.25 | 11.94 | *B* |
| HBP Rosen Striatum M430V2 (Apr05) RMA Clean | 1435807_at | Cdc42 | cell division cycle 42 | ISC; CR |  | 4 | 136.88 | rs4165065 | 2.42 | 12.81 | *D* |
| HBP Rosen Striatum M430V2 (Apr05) RMA Clean | 1428667_at | Maoa | monoamine oxidase A | Cat; APDA | [10,11,12,13,14,15] | X | 16.26 | rs4165065 | 2.21 | 10.67 | *D* |
| HQF Striatum Exon (Feb09) RMA | 4369241 | Mcoln1 | mucolipin 1 | LT; APDA |  | 8 | 3.51 | rs4165065 | 2.09 | 11.45 | *D* |
| HBP Rosen Striatum M430V2 (Apr05) RMA Clean | 1435165_at | Cntn2 | contactin 2 | RCT |  | 1 | 134.41 | rs4165069 | 2.56 | 11.40 | *B* |
| HBP Rosen Striatum M430V2 (Apr05) RMA Clean | 1439927_at | Palm | paralemmin | RCT | [16] | 10 | 79.26 | rs4165065 | 2.48 | 8.37 | *B* |
| HQF Striatum Exon (Feb09) RMA | 4698214 | Akap9 | A kinase (PRKA) anchor protein (yotiao) 9 | RCT | [17,18] | 5 | 4.08 | rs4165069 | 3.55 | 11.79 | *D* |
| HQF Striatum Exon (Feb09) RMA | 4682782 | Sqstm1 | sequestosome 1 | RCT; APDA | [19] | 11 | 50.01 | rs4165069 | 2.26 | 13.24 | *D* |
| HQF Striatum Exon (Feb09) RMA | 4776271 | Napa | N-ethylmaleimide sensitive fusion protein attachment protein alpha | SMP |  | 7 | 16.69 | rs4165069 | 2.01 | 12.92 | *D* |
| HQF Striatum Exon (Feb09) RMA | 4474971 | Pten | phosphatase and tensin homolog | SMP |  | 19 | 32.83 | rs4165069 | 2.22 | 12.00 | *D* |
| HQF Striatum Exon (Feb09) RMA | 5513224 | Myt1l | myelin transcription factor 1-like | TR; APDA | [20] | 12 | 30.46 | rs4165065 | 2.29 | 12.07 | *D* |
| VCU BXD PFC Sal M430 2.0 (Dec06) RMA | 1431749_a_at | Rasgrp1 | RAS guanyl releasing protein 1 | ISC |  | 2 | 117.12 | rs4165069 | 2.97 | 8.39 | *D* |
| VCU BXD PFC Sal M430 2.0 (Dec06) RMA | 1421013_at | Pitpnb | phosphatidylinositol transfer protein, beta | LT |  | 5 | 111.78 | rs4165081 | 1.81 | 8.60 | *D* |
| VCU BXD PFC Sal M430 2.0 (Dec06) RMA | 1423752_at | Ddx47 | DEAD box polypeptide 47 | RT |  | 6 | 134.97 | rs4165081 | 2.89 | 8.72 | *B* |
| VCU BXD PFC Sal M430 2.0 (Dec06) RMA | 1426400_a_at | Capns1 | calpain, small subunit 1 | PT |  | 7 | 30.97 | rs4165069 | 3.18 | 10.08 | *B* |
| VCU BXD PFC Sal M430 2.0 (Dec06) RMA | 1450249_s_at | Kif5a | kinesin family member 5A | T | [21] | 10 | 126.67 | rs4165069 | 1.96 | 9.67 | *D* |
| VCU BXD PFC Sal M430 2.0 (Dec06) RMA | 1428717_at | Scrn1 | secernin 1 | T |  | 6 | 54.46 | rs4165081 | 3.11 | 8.96 | *D* |
| VCU BXD PFC Sal M430 2.0 (Dec06) RMA | 1440639_at | Dlgap1 | discs, large homolog-associated protein 1 | RCT | [22] | 17 | 71.01 | rs4165069 | 1.92 | 8.13 | *B* |
| VCU BXD PFC Sal M430 2.0 (Dec06) RMA | 1452894_at | Elavl4 | ELAV-like 4 (HuD) | SMP; RT | [23,24] | 4 | 109.88 | rs4165081 | 2.04 | 9.61 | *D* |
| VCU BXD PFC Sal M430 2.0 (Dec06) RMA | 1431191_a_at | Syt1 | synaptotagmin 1 | SMP | [25] | 10 | 108.02 | rs4165069 | 2.34 | 10.55 | *D* |
| VCU BXD PFC Sal M430 2.0 (Dec06) RMA | 1450184_s_at | Tef | thyrotroph embryonic factor | TR |  | 15 | 81.65 | rs4165069 | 2.48 | 8.39 | *D* |
| VCU BXD NA Sal M430 2.0 (Oct07) RMA | 1435566_s_at | Araf | v-raf murine sarcoma 3611 viral oncogene homolog | ISC | [26] | X | 20.43 | rs4165065 | 3.07 | 11.57 | *B* |
| VCU BXD NA Sal M430 2.0 (Oct07) RMA | 1424863_a_at | Hipk2 | homeodomain interacting protein kinase 2 | ISC | [27,28] | 6 | 38.65 | rs4165065 | 2.43 | 10.29 | *D* |
| VCU BXD NA Sal M430 2.0 (Oct07) RMA | 1424886_at | Ptprd | protein tyrosine phosphatase, receptor type, D | CR; ISC; APDA | [2] | 4 | 75.59 | rs4165065 | 2.21 | 11.24 | *D* |
| VCU BXD NA Sal M430 2.0 (Oct07) RMA | 1423202_a_at | Ncor1 | nuclear receptor co-repressor 1 | TR; HR |  | 11 | 62.13 | rs4165065 | 2.43 | 11.33 | *D* |
| VCU BXD NA Sal M430 2.0 (Oct07) RMA | 1450350_a_at | Jundm2 | Jun proto-oncogene related gene d2 | TR; HR |  | 12 | 86.98 | rs4165065 | 3.15 | 10.03 | *D* |
| VCU BXD NA Sal M430 2.0 (Oct07) RMA | 1419159_at | Golga3 | golgi autoantigen, golgin subfamily a, 3 | T |  | 5 | 110.65 | rs4165065 | 3.40 | 9.22 | *D* |
| VCU BXD NA Sal M430 2.0 (Oct07) RMA | 1436512_at | Arl7 | ADP-ribosylation factor-like 7 | T; APDA |  | 1 | 90.60 | rs4165065 | 2.75 | 8.90 | *B* |
| VCU BXD NA Sal M430 2.0 (Oct07) RMA | 1447827_x_at | Spop | speckle-type POZ protein | PT |  | 11 | 0.00 | rs4165081 | 1.99 | 10.70 | *D* |
| VCU BXD NA Sal M430 2.0 (Oct07) RMA | 1423055_at | Nsg1 | neuron specific gene family member 1 | RCT | [29,30,31] | 5 | 38.53 | rs4165065 | 3.42 | 12.48 | *B* |
| VCU BXD NA Sal M430 2.0 (Oct07) RMA | 1449878_a_at | Slc12a6 | solute carrier family 12, member 6 | SMP; APDA | [32,33,34,35] | 2 | 112.20 | rs4165065 | 2.66 | 10.66 | *D* |
| **Ref, Literature citation; Chr, Chromosome; Mb, Megabase; LOD, Likelihood of the odds ratio; Expr, Expression; CR, Cytoskeleton regulation; ISC, Intracellular signaling cascade; APDA, Addiction, psychiatric, or neurological disorder associated; RT, RNA processing/transport; PT, Protein processing/transport; SMP, Synaptic maintenance/plasticity; TR, Transcriptional regulation; Cat, Catecholamine regulation; LT, Lipid processing/transport; RCT, Receptor or channel modification/trafficking; T, Transport; HR, Hormone regulation.** | | | | | | | | | | | |

1. Grimbert P, Valanciute A, Audard V, Pawlak A, Le gouvelo S, et al. (2003) Truncation of C-mip (Tc-mip), a new proximal signaling protein, induces c-maf Th2 transcription factor and cytoskeleton reorganization. J Exp Med 198: 797-807.

2. Uhl GR, Liu QR, Drgon T, Johnson C, Walther D, et al. (2008) Molecular genetics of successful smoking cessation: convergent genome-wide association study results. Arch Gen Psychiatry 65: 683-693.

3. Lykidis A, Wang J, Karim MA, Jackowski S (2001) Overexpression of a mammalian ethanolamine-specific kinase accelerates the CDP-ethanolamine pathway. J Biol Chem 276: 2174-2179.

4. Lebeau G, Maher-Laporte M, Topolnik L, Laurent CE, Sossin W, et al. (2008) Staufen1 regulation of protein synthesis-dependent long-term potentiation and synaptic function in hippocampal pyramidal cells. Mol Cell Biol 28: 2896-2907.

5. Vessey JP, Macchi P, Stein JM, Mikl M, Hawker KN, et al. (2008) A loss of function allele for murine Staufen1 leads to impairment of dendritic Staufen1-RNP delivery and dendritic spine morphogenesis. Proc Natl Acad Sci U S A 105: 16374-16379.

6. Ho A, Liu X, Sudhof TC (2008) Deletion of Mint proteins decreases amyloid production in transgenic mouse models of Alzheimer's disease. J Neurosci 28: 14392-14400.

7. Guillaud L, Setou M, Hirokawa N (2003) KIF17 dynamics and regulation of NR2B trafficking in hippocampal neurons. J Neurosci 23: 131-140.

8. Johnson C, Drgon T, Liu QR, Zhang PW, Walther D, et al. (2008) Genome wide association for substance dependence: convergent results from epidemiologic and research volunteer samples. BMC Med Genet 9: 113.

9. Shi Y, Zhao X, Yu L, Tao R, Tang J, et al. (2004) Genetic structure adds power to detect schizophrenia susceptibility at SLIT3 in the Chinese Han population. Genome Res 14: 1345-1349.

10. Huang SY, Lin WW, Wan FJ, Chang AJ, Ko HC, et al. (2007) Monoamine oxidase-A polymorphisms might modify the association between the dopamine D2 receptor gene and alcohol dependence. J Psychiatry Neurosci 32: 185-192.

11. Nakamura K, Sekine Y, Takei N, Iwata Y, Suzuki K, et al. (2009) An association study of monoamine oxidase A (MAOA) gene polymorphism in methamphetamine psychosis. Neurosci Lett 455: 120-123.

12. Kim-Cohen J, Caspi A, Taylor A, Williams B, Newcombe R, et al. (2006) MAOA, maltreatment, and gene-environment interaction predicting children's mental health: new evidence and a meta-analysis. Mol Psychiatry 11: 903-913.

13. Cases O, Seif I, Grimsby J, Gaspar P, Chen K, et al. (1995) Aggressive behavior and altered amounts of brain serotonin and norepinephrine in mice lacking MAOA. Science 268: 1763-1766.

14. Chen K, Cases O, Rebrin I, Wu W, Gallaher TK, et al. (2007) Forebrain-specific expression of monoamine oxidase A reduces neurotransmitter levels, restores the brain structure, and rescues aggressive behavior in monoamine oxidase A-deficient mice. J Biol Chem 282: 115-123.

15. Kim JJ, Shih JC, Chen K, Chen L, Bao S, et al. (1997) Selective enhancement of emotional, but not motor, learning in monoamine oxidase A-deficient mice. Proc Natl Acad Sci U S A 94: 5929-5933.

16. Arstikaitis P, Gauthier-Campbell C, Carolina Gutierrez Herrera R, Huang K, Levinson JN, et al. (2008) Paralemmin-1, a modulator of filopodia induction is required for spine maturation. Mol Biol Cell 19: 2026-2038.

17. Lin JW, Wyszynski M, Madhavan R, Sealock R, Kim JU, et al. (1998) Yotiao, a novel protein of neuromuscular junction and brain that interacts with specific splice variants of NMDA receptor subunit NR1. J Neurosci 18: 2017-2027.

18. Westphal RS, Tavalin SJ, Lin JW, Alto NM, Fraser ID, et al. (1999) Regulation of NMDA receptors by an associated phosphatase-kinase signaling complex. Science 285: 93-96.

19. Jiang J, Parameshwaran K, Seibenhener ML, Kang MG, Suppiramaniam V, et al. (2009) AMPA receptor trafficking and synaptic plasticity require SQSTM1/p62. Hippocampus 19: 392-406.

20. Vrijenhoek T, Buizer-Voskamp JE, van der Stelt I, Strengman E, Sabatti C, et al. (2008) Recurrent CNVs disrupt three candidate genes in schizophrenia patients. Am J Hum Genet 83: 504-510.

21. Xia CH, Roberts EA, Her LS, Liu X, Williams DS, et al. (2003) Abnormal neurofilament transport caused by targeted disruption of neuronal kinesin heavy chain KIF5A. J Cell Biol 161: 55-66.

22. Naisbitt S, Kim E, Weinberg RJ, Rao A, Yang FC, et al. (1997) Characterization of guanylate kinase-associated protein, a postsynaptic density protein at excitatory synapses that interacts directly with postsynaptic density-95/synapse-associated protein 90. J Neurosci 17: 5687-5696.

23. Chung S, Jiang L, Cheng S, Furneaux H (1996) Purification and properties of HuD, a neuronal RNA-binding protein. J Biol Chem 271: 11518-11524.

24. Tiruchinapalli DM, Caron MG, Keene JD (2008) Activity-dependent expression of ELAV/Hu RBPs and neuronal mRNAs in seizure and cocaine brain. J Neurochem 107: 1529-1543.

25. Tang J, Maximov A, Shin OH, Dai H, Rizo J, et al. (2006) A complexin/synaptotagmin 1 switch controls fast synaptic vesicle exocytosis. Cell 126: 1175-1187.

26. Wu X, Noh SJ, Zhou G, Dixon JE, Guan KL (1996) Selective activation of MEK1 but not MEK2 by A-Raf from epidermal growth factor-stimulated Hela cells. J Biol Chem 271: 3265-3271.

27. Kim YH, Choi CY, Lee SJ, Conti MA, Kim Y (1998) Homeodomain-interacting protein kinases, a novel family of co-repressors for homeodomain transcription factors. J Biol Chem 273: 25875-25879.

28. Zhang J, Pho V, Bonasera SJ, Holtzman J, Tang AT, et al. (2007) Essential function of HIPK2 in TGFbeta-dependent survival of midbrain dopamine neurons. Nat Neurosci 10: 77-86.

29. Steiner P, Alberi S, Kulangara K, Yersin A, Sarria JC, et al. (2005) Interactions between NEEP21, GRIP1 and GluR2 regulate sorting and recycling of the glutamate receptor subunit GluR2. Embo J 24: 2873-2884.

30. Steiner P, Sarria JC, Glauser L, Magnin S, Catsicas S, et al. (2002) Modulation of receptor cycling by neuron-enriched endosomal protein of 21 kD. J Cell Biol 157: 1197-1209.

31. Utvik JK, Haglerod C, Mylonakou MN, Holen T, Kropf M, et al. (2009) Neuronal enriched endosomal protein of 21 kDa colocalizes with glutamate receptor subunit GLUR2/3 at the postsynaptic membrane. Neuroscience 158: 96-104.

32. Meyer J, Johannssen K, Freitag CM, Schraut K, Teuber I, et al. (2005) Rare variants of the gene encoding the potassium chloride co-transporter 3 are associated with bipolar disorder. Int J Neuropsychopharmacol 8: 495-504.

33. Howard HC, Mount DB, Rochefort D, Byun N, Dupre N, et al. (2002) The K-Cl cotransporter KCC3 is mutant in a severe peripheral neuropathy associated with agenesis of the corpus callosum. Nat Genet 32: 384-392.

34. Boettger T, Rust MB, Maier H, Seidenbecher T, Schweizer M, et al. (2003) Loss of K-Cl co-transporter KCC3 causes deafness, neurodegeneration and reduced seizure threshold. Embo J 22: 5422-5434.

35. Uyanik G, Elcioglu N, Penzien J, Gross C, Yilmaz Y, et al. (2006) Novel truncating and missense mutations of the KCC3 gene associated with Andermann syndrome. Neurology 66: 1044-1048.
