# Supplementary material for: A Transposon in Comt Generates mRNA Variants and Causes Widespread Expression and Behavioral Differences among Mice
Source: PLoS One. 2010 Aug 17;5(8):e12181. doi: 10.1371/journal.pone.0012181 (PMC2923157; doi:10.1371/journal.pone.0012181)
Supplement: Text S1 — Supplemental methods. (0.07 MB DOC) [file pone.0012181.s009.doc]

# Supplemental Methods

Expression and QTL analysis of Comt using GeneNetwork. Numerous collaborations have resulted in the generation of multiple microarray datasets from many brain and peripheral tissues of inbred and RI strains [1,2,3,4,5,6]. Microarray databases used for analysis of *Comt* expression included: UCHSC BXD Whole Brain M430 2.0 (Nov06) RMA; SJUT Cerebellum mRNA M430 (Mar05) RMA; Hippocampus Consortium M430v2 (Jun06) RMA; HBP Rosen Striatum M430V2 (Apr05) RMA Clean; VCU BXD PFC Sal M430 2.0 (Dec06) RMA; VCU BXD NA Sal M430 2.0 (Oct07) RMA; VCU BXD VTA Sal M430 2.0 (Jun09) RMA; Eye M430v2 (Sep08) RMA; Mouse kidney M430v2 Sex Balanced (Aug06) RMA; UMUTAffy Hippocampus Exon (Feb09) RMA; HQF Striatum Exon (Feb09) RMA. Also used were AKXD NCI Mammary mRNA M430 (July04) RMA [7], Eye AXBXA Illumina V6.2(Oct08) RankInv Beta [8], UCLA CTB6/B6CTF2 Liver (2005) mlratio [9], and UCLA BHF2 Brain (June05) mlratio [10].

In order to ensure an accurate comparison across datasets the Affymetrix platform databases that applied the Robust Multichip Analysis (RMA) normalization method [11] were selected unless otherwise noted. Most Affymetrix databases were standardized for cross-comparison to a mean log2 expression level of 8 and a corresponding standard deviation of ±2 [4] with the exception of the VCU databases. The expression values from the VCU databases were adjusted for all comparisons used in our analysis by adding a factor of 1.5 to each expression value. For reference, all expression levels below six in these databases are considered to be very close to background expression. A detailed description of strain, sex, tissue preparation and microarray method for each individual database is available at [www.genenetwork.org](http://www.genenetwork.org/).

Isolation and sequencing of Comt 3’ UTR mRNA. After DNase treatment, 1 ug of RNA from each strain and the oligo-dT 3’ RACE adaptor (FirstChoice® RLM-RACE Kit from Ambion) were used to perform the reverse transcription (RT) reaction. Target cDNA was amplified by PCR from 1 ul of the RT reaction using a *Comt* custom forward primer (5'-GAA TCC TCT GCA CCC AAG AA-3’) and the 3’ RACE inner primer (5'-CGC GGA TCC GAA TTA ATA CGA CTC ACT ATA GG-3'). PCR was performed using the HotStar HiFidelity Polymerase kit (Qiagen). The predicted size of the PCR product was ~750 bp.

Protein extraction. Protein extraction was performed as follows. Thawed tissue was pre-washed in 0.5 ml pre-cooled PBS followed by a wash in 0.2 ml ice-cold Buffer A (10 mM HEPES, pH7.9, 1.5 mM MgCl2, 10 mM KCl, 0.5 mM DTT). Tissue was disrupted in 400 ul Buffer A for 10-15 s using a Pro200 homogenizer at setting 2 (PRO Scientific). The homogenate was centrifuged at 14,000 rpm at 4 °C for 1 min. The supernatant (soluble portion) was transferred to a new tube and the pellet (nuclear portion) was resuspended in 200ul Buffer C (20 mM HEPES, pH7.9, 25% (V/V) glycerol, 420 mM KCl, 1.5 mM MgCl, 0.2 mM EDTA, 0.5 mM DTT, 0.5 mM phenylmethylsulfonyl fluoride, PMSF). After a 15 min incubation on ice, an equal volume of Buffer D (20 mM HEPES, pH7.9, 20% (V/V) glycerol, 0.2 mM EDTA, 0.5 mM DTT, 0.5 mM PMSF) was added to the resuspended nuclear portion followed by centrifugation for 15 min at 4°C at 10,000 x g. The soluble and nuclear portions were combined together to create a total protein sample and the protein concentration was determined using the Thermo Scientific Pierce BCA Protein Assay Kit (Thermo Scientific). Aliquots were created from each total protein sample and stored at -80°C until use.

Mapping traits to the Comt Locus. Marker rs4165069 (17.577MB) was selected for phenotype mapping because it flanks the chromosome 16 region containing the *Comt* gene (18.407 – 18.427MB). Probe sets mapping to *Comt* were identified using the advanced search option [*mean=(8 20) LRS=(8 999 Chr16 16 20) transLRS=(8 999 10)*] in GeneNetwork in the following databases: HBP Rosen Striatum M430V2 (Apr05) RMA Clean; VCU BXD PFC Sal M430 2.0 (Dec06) RMA; VCU BXD NA Sal M430 2.0 (Oct07) RMA; Hippocampus Consortium M430v2 (Jun06) RMA. Additional probesets mapping to *Comt* were also identified using the UMUTAffy Hipocampus Exon (Feb09) RMA and the HQF Striatum Exon (Feb09) RMA databases and the advanced search option [*mean=(11 20) LRS=(8 999 Chr16 16 20) transLRS=(8 999 10)*].

1. Peirce JL, Li H, Wang J, Manly KF, Hitzemann RJ, et al. (2006) How replicable are mRNA expression QTL? Mamm Genome 17: 643-656.

2. Tapocik JD, Letwin N, Mayo CL, Frank B, Luu T, et al. (2009) Identification of candidate genes and gene networks specifically associated with analgesic tolerance to morphine. J Neurosci 29: 5295-5307.

3. Overall RW, Kempermann G, Peirce J, Lu L, Goldowitz D, et al. (2009) Genetics of the hippocampal transcriptome in mice: a systematic survey and online nerogenomic resource. Frontiers of Neurogenetics 1.

4. Geisert EE, Lu L, Freeman-Anderson NE, Templeton JP, Nassr M, et al. (2009) Gene expression in the mouse eye: an online resource for genetics using 103 strains of mice. Mol Vis 15: 1730-1763.

5. Rosen GD, Pung CJ, Owens CB, Caplow J, Kim H, et al. (2009) Genetic modulation of striatal volume by loci on Chrs 6 and 17 in BXD recombinant inbred mice. Genes Brain Behav 8: 296-308.

6. Saba L, Bhave SV, Grahame N, Bice P, Lapadat R, et al. (2006) Candidate genes and their regulatory elements: alcohol preference and tolerance. Mamm Genome 17: 669-688.

7. Yang H, Crawford N, Lukes L, Finney R, Lancaster M, et al. (2005) Metastasis predictive signature profiles pre-exist in normal tissues. Clin Exp Metastasis 22: 593-603.

8. Whitney IE, Raven MA, Ciobanu DC, Williams RW, Reese BE (2009) Multiple genes on chromosome 7 regulate dopaminergic amacrine cell number in the mouse retina. Invest Ophthalmol Vis Sci 50: 1996-2003.

9. Schadt EE, Molony C, Chudin E, Hao K, Yang X, et al. (2008) Mapping the genetic architecture of gene expression in human liver. PLoS Biol 6: e107.

10. Yang X, Schadt EE, Wang S, Wang H, Arnold AP, et al. (2006) Tissue-specific expression and regulation of sexually dimorphic genes in mice. Genome Res 16: 995-1004.

11. Irizarry RA, Hobbs B, Collin F, Beazer-Barclay YD, Antonellis KJ, et al. (2003) Exploration, normalization, and summaries of high density oligonucleotide array probe level data. Biostatistics 4: 249-264.
